# Optimisation of low-level light therapy (lllt) parameters and evaluation of its immunomodulatory effect on fibroblasts

**DOI:** 10.1007/s10103-026-04873-w

**Published:** 2026-05-09

**Authors:** Anna Ścisłowska-Czarnecka, Amelia Lizak, Aleksandra Matuła, Aneta Bac, Joanna Homa, Magdalena Chadzińska, Beata Stenka

**Affiliations:** 1 Department of Applied Cosmetology, University of Physical Culture, Kraków, Poland; 2 Department of Evolutionary Immunology, Institute of Zoology and Biomedical Research, Jagiellonian University, Kraków, Poland; 3 Department of Applied Cosmetology, University of Physical Education and Sport, Gdańsk, Poland

**Keywords:** LLLT, Fibroblasts, Cytokines, MMP, Oxidative-antioxidant potential

## Abstract

Low-level laser therapy (LLLT) is being increasingly applied in regenerative medicine due to its ability to modulate cellular metabolism and support repair processes. However, the effect of various exposure parameters on fibroblast activity still does not remain fully understood. The study aim was to evaluate the effect of selected LLLT parameters (808 nm, 100 and 200 mW, 2 and 10 J/cm², pulsed beam) on the biological activity of fibroblasts in vitro. Fibroblasts were exposed to the established exposure parameters, and then the number of adherent and metabolically active cells, their migration, adenylate kinase (AK) as well as nitric oxide (NO) levels, cytokine secretion, metalloproteinase (MMP-2, MMP-9) activity, and oxidative-antioxidant potential were assessed. The most favourable effects were achieved with 200 mW; 2 J/cm² and 100 m; 10 J/cm² exposure parameters. In these conditions, increased number of adherent and metabolically active cells, and elevated migration were observed. Moreover, in this condition decreased AK release and NO secretion were found. Furthermore, LLLT reduced in fibroblasts interleukin 6 (IL-6) and interleukin 8 (IL-8) secretion as well as affected the activity of metalloproteinases, what corresponded with subsequent wound healing process. Additionally, a beneficial effect on maintaining the oxidative-antioxidant balance in fibroblasts was noted. In summary, the effects of LLLT on fibroblasts depend on the applied irradiation parameters, and appropriately selected LLLT treatment (200 mW; 2 J/cm² and 100 mW; 10 J/cm²,) stabilise fibroblast metabolism, limits their proinflammatory response and promotes extracellular matrix remodelling (ECM). These results confirm the potential of LLLT as an effective method supporting regenerative processes in the treatment of tissue damage.

**Supplementary Information:**

The online version contains supplementary material available at 10.1007/s10103-026-04873-w.

## Introduction

The healing process occurs in three overlapping phases: inflammatory, proliferative and remodelling [1]. Their proper course determines effective restoration of the skin’s structural and functional integrity. Disturbances in the balance between these stages represent a significant clinical problem and may lead to the development of chronic wounds. Treating such wounds remains a therapeutic challenge due to persistent inflammation and abnormal extracellular matrix (ECM) remodelling [2–4]. Although fibroblasts have traditionally been viewed primarily as cells responsible for maintaining skin structure, an increasing number of studies highlight their regulatory role in the wound‑healing process [5–7]. During the inflammatory phase, fibroblasts secrete proinflammatory cytokines, including tumor necrosis factor α (TNF-α), interleukin 1 (IL-1) and chemokines, such as CCL2 and CXCL1, which enhance leukocyte recruitment to the injury site [1, 8, 9]. In the proliferative phase, fibroblasts interact with macrophages, modulating their transition from the M1 (proinflammatory) to M2 (anti-inflammatory and reparative) phenotype, which enables inflammatory response quenching and initiation of regenerative processes [10]. During the remodelling phase, these cells exhibit immunosuppressive activity by secreting anti-inflammatory cytokines such as transforming growth factor β (TGF-β) and interleukin 10 (IL-10). Furthermore, fibroblasts are the main source of matrix metalloproteinases (MMPs), which play a key role in regulating ECM remodelling [11].

The dysfunction of fibroblasts in each healing phases may additionally disrupt the repair process, leading to delayed regeneration or pathological scarring, observed, among others, in ulcers, pressure sores or chronic dermatoses [1, 12]. The complexity of these mechanisms means that in addition to pharmacological therapies other methods are sought to support physiological repair processes. One of them is low-level laser therapy (LLLT), which is credited with the ability to photobiomodulate (PBM) cell activity. Mechanistically, the action of PBM is primarily associated with the absorption of radiation by mitochondrial cytochrome c oxidase (CCO), which leads to changes in ATP and reactive oxygen species (ROS) production and in the activation of intracellular signalling pathways. In numerous in vitro studies, it has been shown that LLLT can activate several intracellular signalling pathways, including protein kinase B (Akt), extracellular signal-regulated kinase (ERK), and c-Jun N-terminal kinase (JNK), which are associated with cell proliferation, migration, secretion of cytokines and metalloproteinases (MMPs), and may influence the oxidative-antioxidant balance of cells [13, 14]. Consequently, LLLT can impact the healing process by modulating the duration and intensity of the inflammatory and proliferative phases of this process, as well as by supporting extracellular matrix remodelling and initiation of repair processes [15].

However, not all studies confirm the beneficial effects of LLLT and discrepancies in the results are primarily attributed to differences in the irradiation parameters used, such as wavelength, power, energy dose or exposure time. This parameters determine the nature and intensity of the cellular response, often biphasic in nature, which further translates into ambiguous research results [16, 17]. In some of them, unsignificant changes in cell functioning were observed after exposure to laser radiation, and in some reports, adverse effects are indicated, such as inhibition of cell proliferation and migration as well as increase of the oxidative stress [18, 19]. The significance of proper parameter selection was also confirmed in our previous studies conducted on macrophages, in which we found that low-energy laser irradiation modulates macrophage activity in a manner dependent on the applied settings, leading to stimulating or cytotoxic effects [20]. The varied effects observed depending on the used LLLT parameters and cell type indicate the need for further research aimed at precisely defining the irradiation conditions enabling effective and safe stimulation of regenerative processes.

Therefore, the aim of this study was to determine LLLT parameters which optimise the fibroblast response and to assess their impact on the inflammatory response, ECM remodelling and oxidative-antioxidant balance, all important for the regulation of the repair processes in the skin.

## Materials and methods

### Fibroblast culture conditions

Human fibroblast cell line Hs680.Tr (ATCC, USA) was cultured in an DMEM medium (Lonza, USA), supplemented with 10% FBS (Gibco, USA) and a 1% antibiotic solution: penicillin and streptomycin (Sigma-Aldrich, Germany). Fibroblasts from passages 5–6 were used for the experiment. Cells at a concentration of 0.01 million cells/ml were plated into wells of a 24-well culture plate (Nest SB, USA). The culture was maintained in an incubator (MCO-18AC PhCbi, UK) for 3 and 5 days, respectively, in an atmosphere of 5% CO2 and at 37 °C. The culture medium was not changed during the experimental period. In order to determine the secretory activity of fibroblasts, after a specified culture time of 3 and 5 days, respectively, the cell culture supernatant was collected and frozen (at −20 °C) for later assays.

### Irradiation with low-energy laser radiation

The fibroblasts were irradiated with the PhysioGo 400 C (ASTAR, Poland), a low-energy laser generating electromagnetic radiation within the infrared range at a wavelength of 808 nm, a power of 100 mW or 200 mW, and a radiation dose of 2–10 J/cm^2^/cell well. The laser beam was applied in a pulsed (I) manner (frequency of 100 Hz with a 50% duty cycle). During light exposure, no statistically significant temperature increase was noted in the culture medium. The mean temperature change was ΔT = 0.4 ± 0.2 °C, compared to the baseline value (37 ± 0.2 °C). Irradiation was performed on the irradiated surface at a perpendicular angle using the non-contact method at a minimum distance of 1 cm from the cells (height of the plate well).

**Fig. 1:**
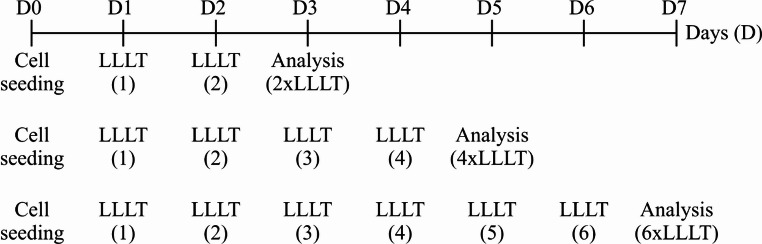
Schematic representation of the experimental design and irradiation timeline. Fibroblasts were seeded on the plate on day 0 (D0) and the first low-level laser therapy (LLLT) exposure was performed 24 h later (day 1, D1). Cells were irradiated once per day with an 808 nm infrared laser (100 mW or 200 mW; 2–10 J/cm² per well) operating in pulsed mode (100 Hz frequency, 50% duty cycle). Irradiation was performed once per day for 2, 4, or 6 consecutive days, corresponding to LLLT sessions on days D1 and D2 (2×LLLT), days D1–D4 (4×LLLT), or days D1–D6 (6×LLLT), respectively. Cellular and biochemical analyses were performed on the day following the final irradiation, corresponding to day 3 (D3; 2×LLLT), day 5 (D5; 4×LLLT) and day 7 (D7; 6×LLLT). LLLT (low-level laser therapy); (D) - day of culture; 2×LLLT, 4×LLLT, 6×LLLT - number of consecutive irradiation sessions

Cells were seeded on the plates on day 0 and for the first time irradiated 24 h later (day 1). Irradiation was performed once per day for 2, 4 or 6 consecutive days. Cellular analyses were conducted on the next day post the final irradiation (days 3, 5 and 7 of the culture) (Fig. 1). The culture medium was not changed during the experimental period. After termination of the culture, both cells and supernatants were collected from the cell culture and were used for further biological assays.

#### Stages of the study

The research was conducted in two stages.

Stage 1 of the study aimed to determine the LLLT parameters most favourably influencing the biological activity of fibroblasts: (i) number of adherent cells, (ii) number of metabolically active cells, (iii) cell migration/proliferation, (iv) adenylate kinase (AK) release and (v) nitric oxide (NO) secretion.

Stage 2 of the study was aimed to assess the potential immunomodulatory effect of LLLT, measured as: (i) the levels of pro- and anti-inflammatory cytokines secreted from fibroblasts, (ii) activity of matrix metalloproteinases (MMPs) and (iii) cellular oxidative-antioxidant potential.

### Cell number (CV test)

The crystal violet (CV) assay was used to assess the total number of adherent cells, reflecting the combined effects of cell adhesion and proliferation. Cells adhering to the substrate were fixed for 5 min in 2% paraformaldehyde (PF) (Sigma**-**Aldrich, Germany), stained for 5 min with 0.5% crystal violet dissolved in H_2_O (Sigma**-**Aldrich, Germany), and then washed 3 × 1 min with water. The dye absorbed by the cells was extracted by adding 0.5 ml of 100% methanol (Linegal Chemicals, Poland) to each culture well. The optical density (O.D.) of the fluid was measured at 570 nm using the FLUOstar Omega reader (BMG Labtech, Germany).

### Fibroblast migration/proliferation (wound healing assay)

The wound healing assay was used to evaluate the combined effect of fibroblast migration and proliferation by measuring gap closure over time. Fibroblasts were cultured in 24-well plates equipped with Culture-Insert 2 Well 24 inserts (ibidi GmbH, Martinsried, Germany), providing a defined 500 μm cell-free gap. The inserts are made of biocompatible silicone, which allows firm adhesion to the plate surface without the use of additional substrates [21]. Cells were seeded at a density of 0.01 million cells/ml and incubated with the inserts overnight under standard culture conditions (37 °C, 5% CO₂). After incubation, the inserts were removed and the cells were rinsed with culture medium to eliminate non-adherent cells. Subsequently, 1 ml of fresh culture medium was added to each well. Cells in the experimental groups were irradiated according to the applied parameters, whereas the control group (CTR) was not irradiated.

Photographic documentation was performed immediately after insert removal (day 0) and on days 3 and 5 post removal using an inverted microscope (Motic AE-2000T) at 10× magnification, equipped with a Moticam-BTU8 camera (Motic Europe, Spain). All images were acquired using identical microscope settings.

Image analysis was performed using ImageJ software (NIH, Bethesda, MD, USA). The cell-free area was manually delineated as a region of interest (ROI), and measured in pixels. The percentage of wound closure (%) was calculated using the following formula:

$\:\mathrm{Wound\:closure\:(\%)}=\frac{{A}_{0}-{A}_{t}}{{A}_{0}}\times\:100$where $\:{A}_{0}$represents the mean wound area on day 0 and $\:{A}_{t}$represents the wound area on day 3 or 5.

### ATP-dependent metabolic active fibroblast number (ViaLight assay)

To determine the number of metabolically active cells a ready-made assay with the ViaLight reagent kit (Lonza, Switzerland) was used according to the procedure provided by the manufacturer. Briefly. 200 µl of Cell Lysis Reagent was added to the wells containing cells and 600 µl of supernatant. After 10 min of incubation, 200 µl of the supernatant-lyser mixture was transferred to a white 96-well plate (Nest SB, USA), and 200 µl of AMR PLUS reagent was added. After 2 min, the amount of emitted radiation was determined using the FLUOstar Omega reader (BMG Labtech, Germany).

### Level of released adenylate kinase AK (ToxiLight test)

To determined cell membrane damage and cytotoxicity, manifested as adenylate kinase (AK) release, the ToxiLight assay was used. AK levels were determined by bioluminescence quantification using the Toxilight reagent kit according to manufacturer protocol (Lonza, Switzerland). Briefly, 20 µl of the cell culture supernatant was collected and transferred to a white 96-well plate (Nest SB, USA). Then, 100 µl of AK Detection Reagent solution was added to each well. After 5 min of incubation, luminescence was read using a FLUOstar Omega reader (BMG Labtech, Germany).

### Level of NO secretion (Griess test)

To determine the nitric oxide (NO) release into cell culture supernatants the Griess assay was used. Cell supernatants (100 µl) were transferred to the wells of a 96-well plate (Nest SB, USA), and 100 µl of a reagent mixture (Sigma-Aldrich, Germany) containing Griess A (1% sulphanilamide in 5% phosphate acid) and B (0.1% naphthylenediamine in H2O), mixed at a 1:1 ratio, was added. After five minutes, the optical density (O.D.) of the fluid was read at 540 nm using the FLUOstar Omega reader (BMG Labtech, Germany), and then these values ​​were converted to NO concentration based on a calibration curve prepared for sodium nitrite.

### Level of secreted cytokines

Cytokine levels in cell culture supernatants were measured via flow cytometry using Flex Set kits (CBA, BD Biosciences). The entire assay procedure, all measurements and analyses were performed according to the instructions included in the cytokine assay kit of the Beckman Coulter flow cytometer (Life Science, USA). The Humane Inflammation Kit (BD Biosciences, USA) was used, which allows for the simultaneous measurement of six cytokine levels: interleukin (IL-1β), interleukin 6 (IL-6), interleukin 8 (IL-8), interleukin 10 (IL-10), interleukin 12p70 (IL-12p70) and tumour necrosis factor (TNF-α). Data analyses and cytokine concentrations were performed in Microsoft Excel using standard curves generated from serial dilutions of the standard as described previously [22].

### Level of secreted metalloproteinases

Gelatin zymography was used to evaluate the activity of matrix metalloproteinases (MMPs) - markers of extracellular matrix remodelling. Using this modified electrophoretic method, proteolytic enzyme activity can be assessed, as its substrate (gelatin) is incorporated into a polyacrylamide gel containing sodium dodecyl sulphate (SDS). This method allows for the differentiation and quantification of pro-MMP-2, pro-MMP-9 and the active forms of MMP-2 and MMP-9. A detailed zymography protocol has been previously described [20].

### Measurement of oxidation potential (PerOx test – TOS/TOC)

The PerOx assay was used to determine total oxidative status (TOS/TOC) of cells. Total oxidative capacity (TOS) of the cells was determined by measuring lipid peroxide levels according to the instructions for use provided in the PerOx (TOS/TOC) kit (Immunodiagnostik AG, Bensheim, Germany). The detailed protocol has been previously described [23].

### Measurement of antioxidant potential (ImAnOx test – TAS/TAC)

The ImAnOx assay was used to determine total antioxidant status (TAS/TAC). Total Antioxidant Status of the cells was determined through the reaction of antioxidants with a known amount of exogenous hydrogen peroxide (H_2_O_2_), according to the ImAnOx (TAS/TAC) kit protocol (Immunodiagnostik AG, Bensheim, Germany). The detailed protocol has been previously described [23].

###  Statistical analysis

Data are presented as means and standard errors. Differences between the control and study groups were determined using the Student’s *t*-test when the parametric test assumptions were met, and the non-parametric Mann-Whitney U test or the Kruskal–Wallis test when the assumptions were not. Differences between the control and study groups were compared using one-way analysis of variance (ANOVA), followed by post-hoc assessment using Tukey’s test. A significance level of *p* ≤ 0.05 was adopted in the analyses. Microsoft Excel 2007 and GraphPad 7 (GraphPad Software, San Diego, CA, USA) were used for statistical and graphical analysis of the results.

## Results

### Selection of optimal parameters for irradiation of fibroblasts with low-energy laser radiation

**Fig. 2:**
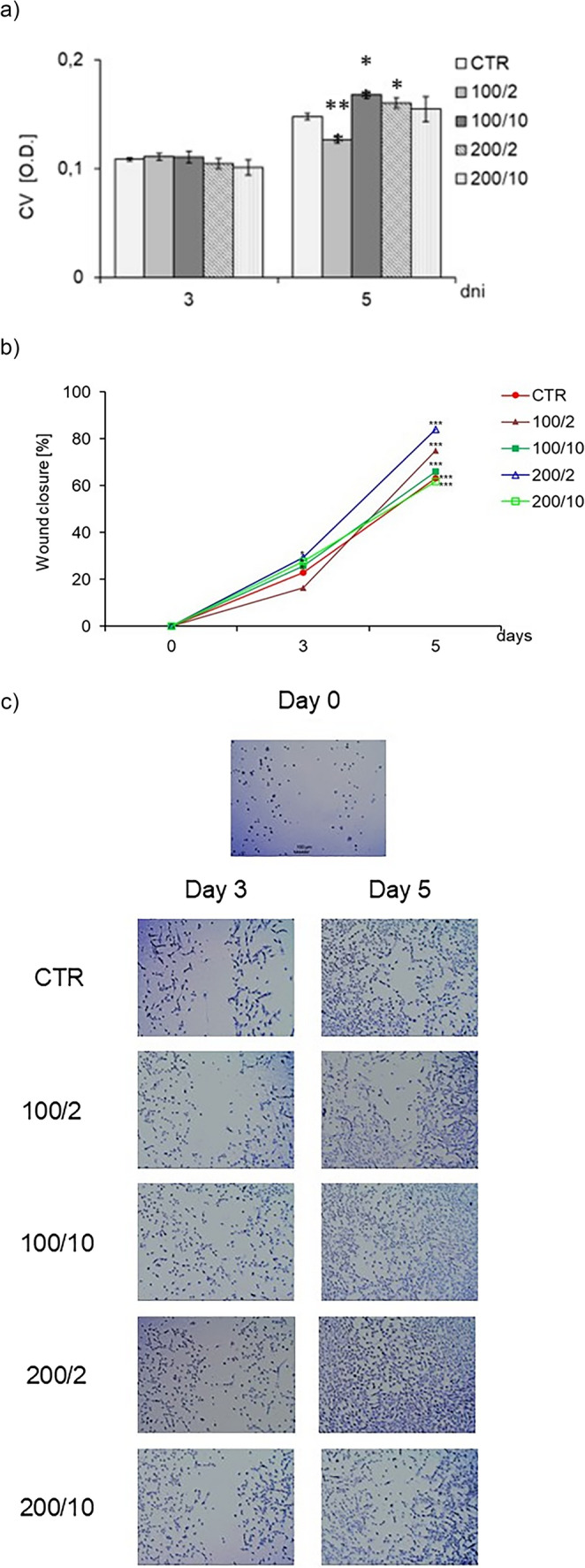
Effect of pulsed laser beam (frequency 100 Hz, 50% duty cycle) at a wavelength of 808 nm on the number of stained with crystal violet (CV) adhered fibroblasts (Figure 2a), quantitative analysis of the wound closure (Figure 2b) and representative images illustrating wound closure (Figure 2c) of the Hs680Tr. cell line. Cells were cultured for a specified number of days and irradiated with a laser at powers of 100 or 200 mW and radiation doses of 2 or 10 J/cm^2^/cell well. On subsequent days of the experiment (3 and 5), cells were stained with crystal violet. Analysis migration was conducted under a light inverted microscope at a total magnification 40x (objective lens magnification (10x) x eyepiece magnification (4x), scale bar 100m. Wound healing was quantified as the percentage of wound closure relative to the initial gap area measured on day 0. O.D. - optical density was measured at 570 nm. Results are presented as means ± SE from three independent experiments. *, ** - differences between cells irradiated with laser of different parameters and cells non-irradiated (CTR), (* *p*<0.05,** *p*<0.01)

On subsequent days of the experiment, fibroblasts in the control, non-irradiated group (CTR group) divided, causing an increase in the number of adherent cells compared to day 3 of their culture (Fig. 1a). Four exposures of a 100 mW laser beam at a dose of 10 J/cm² (100/10 group) and a 200 mW laser beam at a dose of 2 J/cm² (200/2 group) resulted in increased number of adherent fibroblasts on day 5 of cell culturing, compared to the non-irradiated control group (CTR group) (Fig. 2a). A decrease of the number of adherent cells was observed for fibroblasts exposed to a 100 mW laser beam four times at a dose of 2 J/cm² (100/2 group). However, irradiation (four times) with a beam power of 200 mW and a dose of 10 J/cm² (200/10 group) did not affect the number of adherent fibroblasts, compared to the control, non-irradiated group (CTR group) (Fig. 2a).

The degree of wound closure was expressed as the percentage of gap closure relative to day 0. On day 3 of the experiment, a moderate level of wound closure was observed in all examined groups. In the control group, no significant changes were detected compared to baseline. Significantly greater gap closure relative to day 0 was observed in the 200/2 and 200/10 groups. In the 100/10 group, only a tendency towards increased wound closure was noted, whereas in the 100/2 group the wound closure was similar to control group (Fig. 2b).

On day 5, all groups, including the control, demonstrated a significant increase in wound closure compared to day 0. The most pronounced effect was observed in the 200/2 group. A high level of gap closure was also detected in the 100/2 group, whereas the 100/10 group showed greater variability in response (Fig. 1b). Representative images illustrating gap closure are presented in Fig. 2c.

**Fig. 3:**
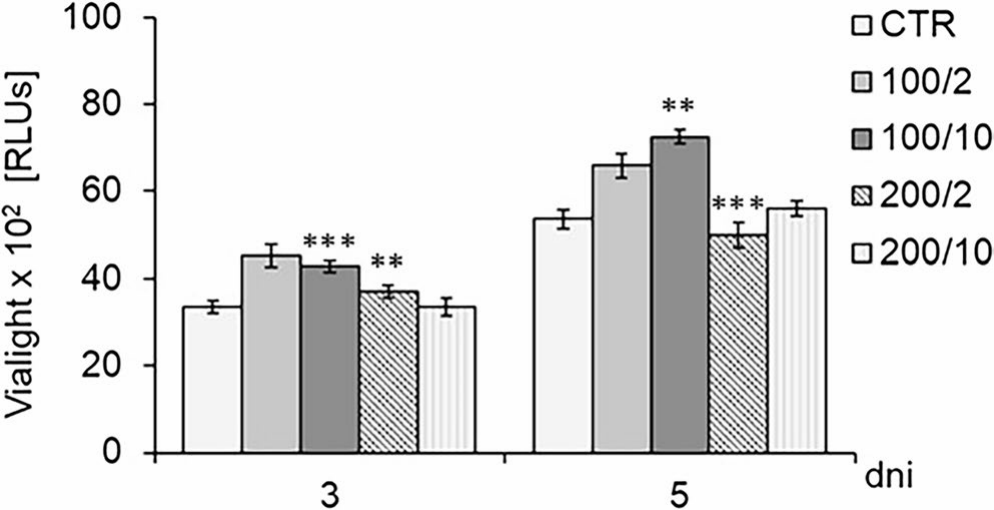
Effect of pulsed laser beam irradiation (frequency 100 Hz, 50% duty cycle) at a wavelength of 808 nm on the number of metabolically active Hs680Tr fibroblasts, . Cells were cultured for a specified number of days and irradiated with a laser at powers of 100 or 200 mW and radiation doses of 2 or 10 J/cm² per well. ATP-dependent luminescence was measured on days 3 and 5 of the experiment. RLUs – relative light units (luminescence units). Results are presented as means ± SE from three independent experiments.***– differences between cells irradiated with laser of different parameters and non-irradiated control cells (CTR), ( *p*<0.01,*** *p*<0.001)

Irradiation of the fibroblasts with a 100 mW beam of radiation and a dose of 10 J/cm² (100/10 group), and with a 200 mW beam of radiation and a dose of 2 J/cm² (200/2 group) resulted in an increase in the number of metabolically active cells on day 5 of the culture. However, irradiation using a 100 mW beam of radiation and a dose of 2 J/cm² (100/2 group), and with a 200 mW beam of radiation and a dose of 10 J/cm² (200/10 group) had no significant effect on the number of metabolically active fibroblasts compared to the control group (CTR) (Fig. 3).

**Fig. 4:**
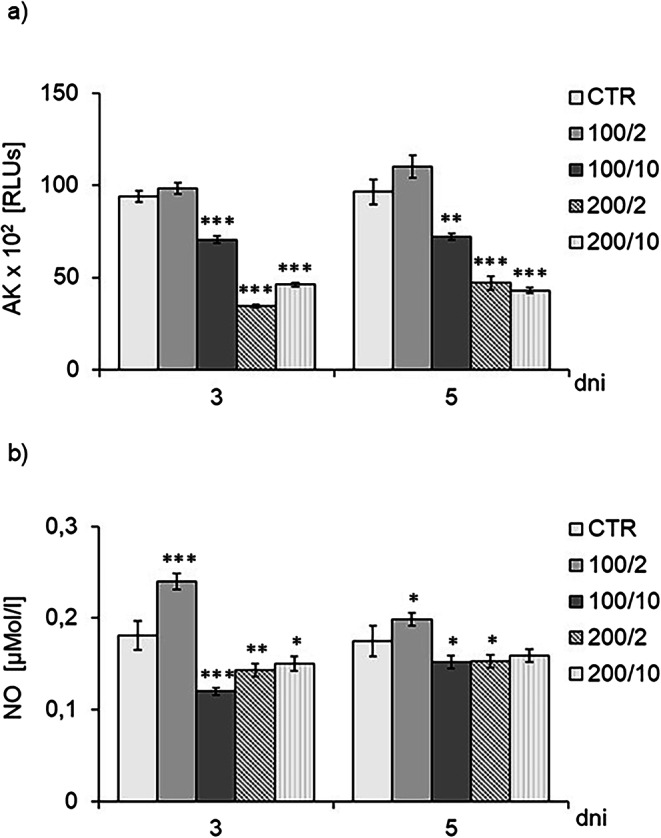
Effect of pulsed laser beam irradiation (frequency 100 Hz, 50% duty cycle) at a wavelength of 808 nm on the levels of the adenylyl kinase (AK) (Figure 4a) and nitric oxide (NO) (Fig. 4b) release levels by fibroblasts of the Hs680Tr. cell line. Cells were cultured for a specified number of days, irradiated with a laser at powers of 100 or 200 mW and radiation doses of 2 or 10 J/cm2/well with cells. AK and NO levels were measured on the next 3 and 5 days of the experiment. RLUs - luminometer flux unit RLUs - luminometer flux unit. O.D. - optical density was measured at 540 nm. Results are presented as means ± SE from three independent experiments. *, **,*** - differences between cells irradiated with laser of different parameters and cells non-irradiated (CTR), (*p<0.05,** *p*<0.01,*** *p*<0.001)

Regardless of the number of irradiations, at the studied time points (third and fifth days of the culture), a decrease in AK release was observed by cells exposed to a 100 mW laser beam with a dose of 10 J/cm² (100/10 group) and to a 200 mW laser beam with doses of 2 and 10 J/cm² (200/2 and 200/10 groups) compared to the control group (CTR) which was not exposed to irradiation (Fig. 3a). In contrast, irradiation of cells with a 100 mW laser beam and a dose of 2 J/cm² (100/2 group) had no effect on AK release, compared to the control group (Fig. 4a).

Irradiation of the fibroblasts with a 100 mW radiation beam and a dose of 2 J/cm^2^ (100/2 group) twice and four times resulted in increased NO secretion compared to the control cell group (CTR group) (Fig. 4b). In turn, irradiation of cells with a 100 mW radiation beam and a dose of 10 J/cm^2^ (100/10 group) twice and with a 200 mW radiation beam and a dose of 2 J/cm^2^, caused a decrease in NO secretion on days 3 and 5 of cell culturing. Irradiation of cells with a 200 mW radiation beam and a dose of 10 J/cm^2^ (200/10 group) demonstrated a decrease in NO secretion by fibroblasts only on day 3 of the culture, compared to the group of non-irradiated cells (Fig. 4b).

### Effect of LLLT on the secretion of cytokines, activity of MMPs and oxidative-antioxidant balance of fibroblasts irradiated with laser beam at 100 mW power and dose of 10 J/cm² (100/10 group) and with laser beam at 200 mW power and dose of 2 J/cm² (200/2 group)

**Fig. 5:**
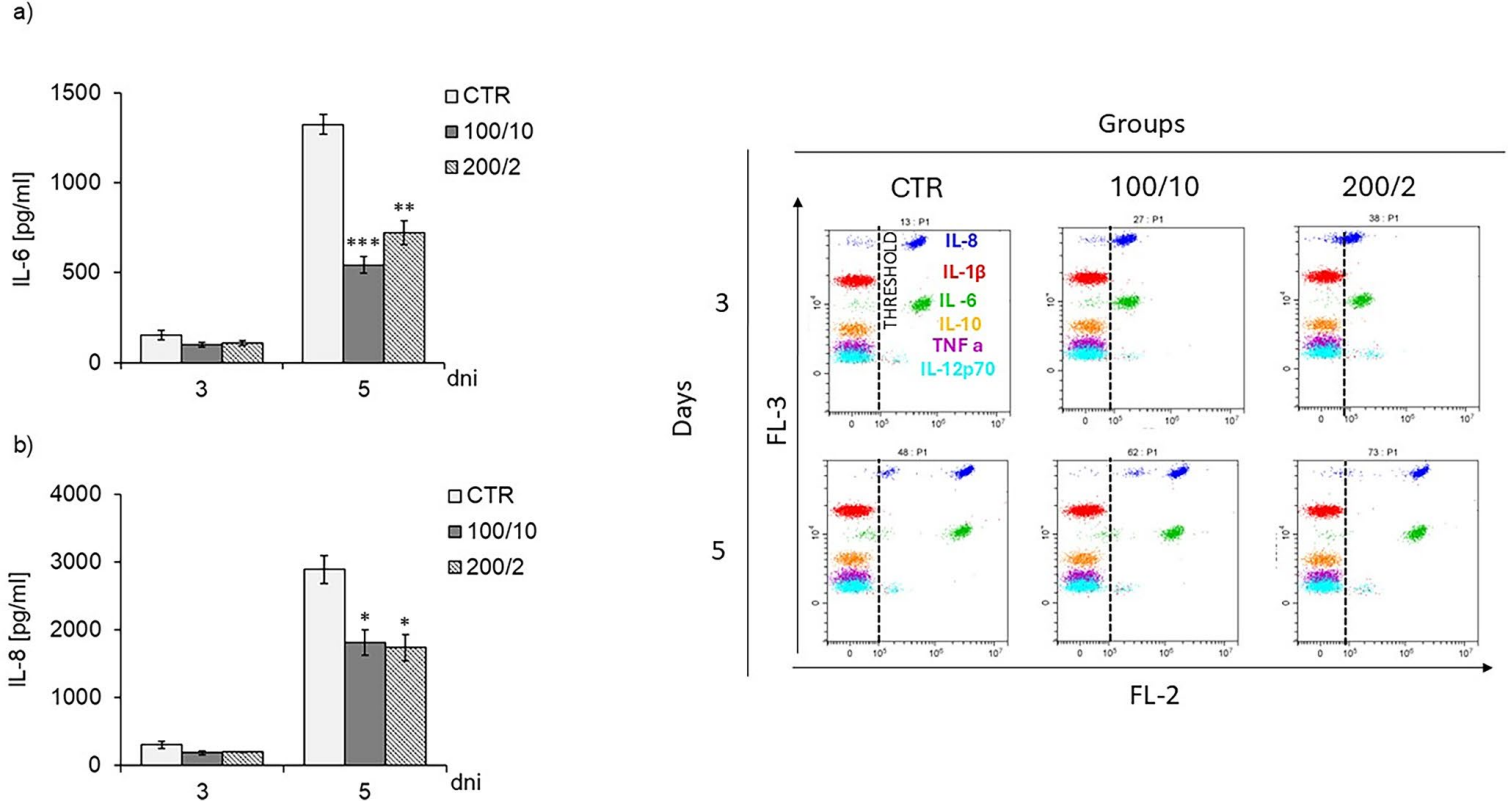
Effect of pulsed laser beam irradiation (frequency 100 Hz, 50% duty cycle) at a wavelength of 808 nm on the levels of the cytokines IL-6 (Figure5a), IL-8 (Figure 5b) secreted by fibroblasts of the Hs680Tr. cell line and example/representative dot plots of cytometric analysis of fluorescently labelled cytokines: IL-6, IL-8 (Figure 5c). Cells were cultured for a specified number of days, irradiated with a laser at powers of 100 mW and radiation doses of 10 J/cm^2^/well with cells or 200 mW and radiation doses of 2 J/cm^2^/well with cells. Cytokine levels were measured on the next 3 and 5 days of the experiment. The remaining parameters are shown as means ± SE calculated from 2-3 representative samples from three independent experiments. *,**, *** - differences between cells irradiated with laser of different parameters and cells non-irradiated (CTR), (* *p*<0.05,** *p*<0.01,*** *p*<0.001)

In the case of the tested cytokines, regardless of the LLLT parameters applied, four-fold laser irradiation resulted in a decrease in the secretion of IL-6 and IL-8 on the fifth days of culturing (Fig. 5a, b). With regard to the remaining cytokines: IL-1β, IL-10, IL-12p70 and TNF-α, their presence was not detected in any of the tested groups, regardless of the implemented irradiation parameters and the tested time point (groups 100/10, 200/2 and CTR), (Fig. 5a, b).

Two exposures of fibroblasts to a 100 mW laser beam and a dose of 10 J/cm² (100/10 group) resulted in an increase in pro-MMP-9 and its dimeric form, as well as MMP-9 dimers on day 3 of the culture in comparison to the non-irradiated control group (CTR group). The further four exposures of fibroblasts to these parameters caused an increase in MMP-2 activity and a decrease in pro-MMP-9 on day 5 of culturing (Table 1). Furthermore, exposure of fibroblasts to a 200 mW laser beam and a dose of 2 J/cm² (200/2 group) increased the pro-MMP-9 and MMP-9 dimers on day 5 of the culture, compared to the control group (CTR group) (Table 1).

**Table 1:**
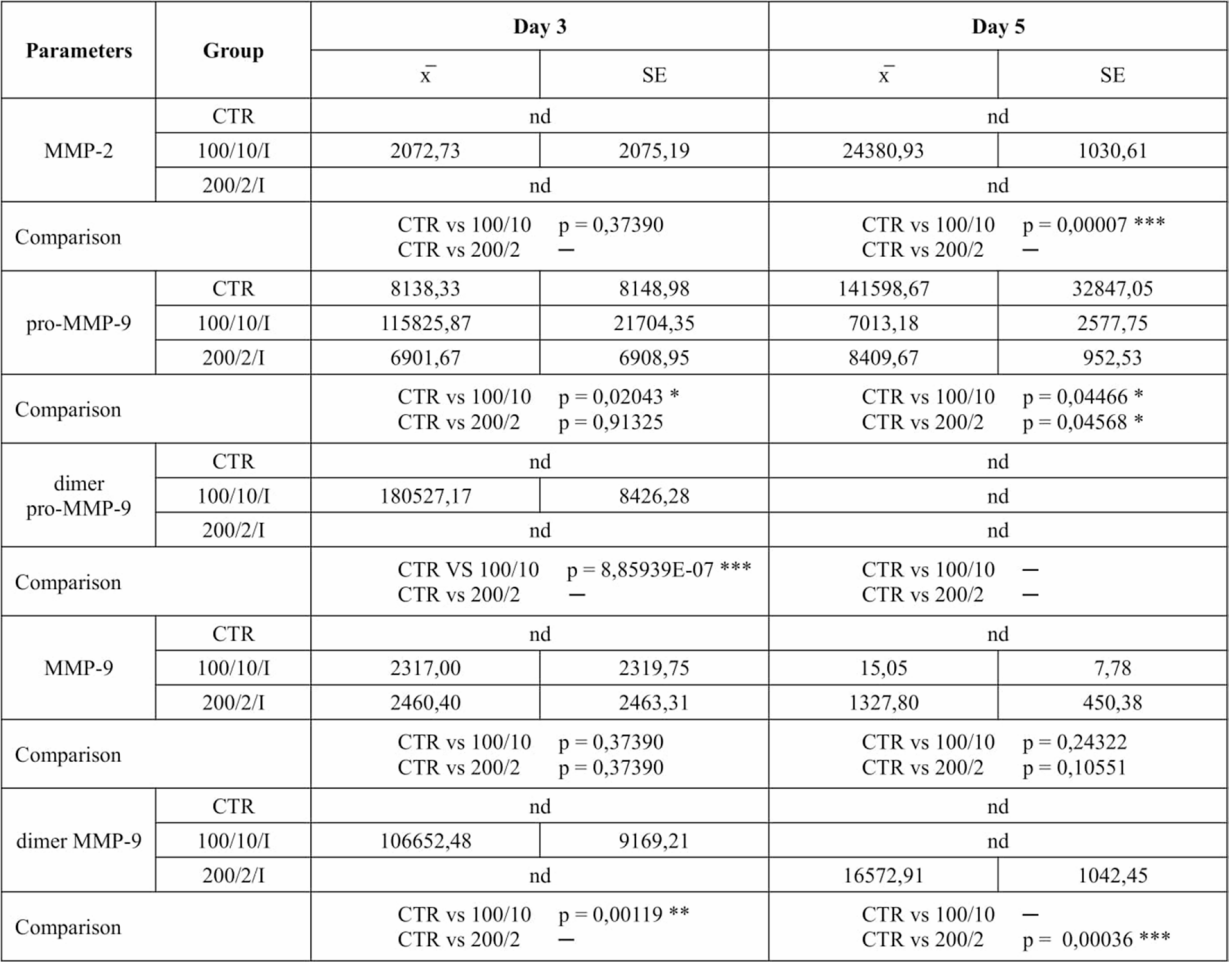
The effect of LLLT on the level of MMPs secreted by fibroblasts of the Hs680Tr. cell line

Cells were cultured for a specified number of days and irradiated with a pulsed laser beam (frequency 100 Hz, 50% duty cycle) at a wavelength of 808 nm, with a power output of 100 or 200 mW and energy doses of 2–10 J/cm² per well containing cells (groups: 100/2, 100/10, 200/2, 200/10). MMP were determined on the following days of the experiment: 3 and 5 days. The average areas of the peaks corresponding to MMP-2, pro-MMP9 dimer pro-MMP-9, MMP-9 and dimer MMP-9 in the samples were calculated and expressed as raw mean values (raw volume). For quantitative analysis, one representative sample per experimental group from each of the three independent experiments was analysed. Data are presented as mean ± SE; nd – not detected (below the detection threshold of the method); values below detection were treated as 0 for statistical analysis. *, **, *** - differences between cells irradiated with laser of different parameters and cells non-irradiated (CTR), (* *p* < 0.05, ** *p* < 0.01, *** *p* < 0.001).

**Fig. 6:**
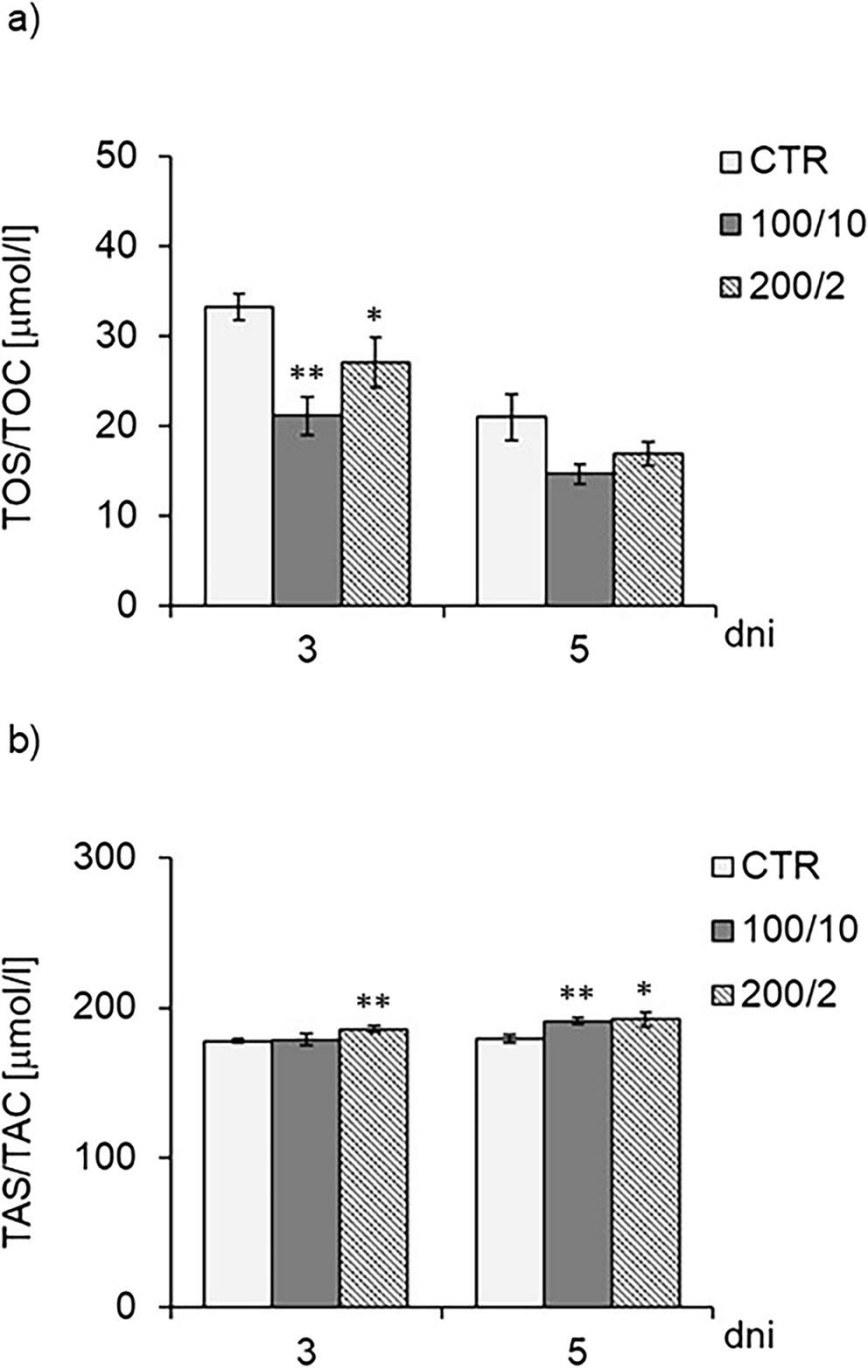
Effect of irradiation with a pulsed laser beam (frequency 100 Hz, 50% duty cycle) at a wavelength of 808 nm on the oxidative (Figure 6a) and antioxidant (Figure 6b) potential of Hs680Tr fibroblasts. Cells were cultured for a specified number of days, irradiated with a laser at powers of 100 mW and radiation doses of 10 J/cm^2^/well with cells or 200 mW and radiation doses of 2 J/cm^2^/well with cells. Oxidative potential (TOS/TOC) and antioxidant potential (TAS/TAC) were determined on the following days of the experiment: 3 and 5 days. Mean values ± SEM. *, ** - differences between cells irradiated with laser of different parameters and cells non-irradiated (CTR), (* *p*<0.05,** *p*<0.01)

The measurement of the fibroblasts’ oxidative potential showed that two- and four-fold irradiation of cells with a radiation beam of 100 mW and a dose of 10 J/cm² (100/10 group) caused a decrease in the oxidative potential of cells (TOS/TOC) on culture days 3 and 5, and an increase in the antioxidant potential (TAS/TAC) on day 5 of the culture, compared to the non-irradiated control (CTR group), (Fig. 6a, b). In the case of cell irradiation with a radiation beam of 200 mW and a dose of 2 J/cm² (200/2 group), an increase in the antioxidant potential was demonstrated as a result of two- and four-fold irradiation of cells (on days 3 and 5 of fibroblast culture, respectively), compared to the control, non-irradiated cells (200/2 group), (Fig. 6a, b).

## Discussion

LLLT may support physiological repair processes by activating cells involved in wound healing, thereby promoting tissue regeneration. However, the effectiveness of this intervention depends on the precise selection of irradiation parameters, such as wavelength, power, energy dose, and emission mode, which determine the nature of the cellular response and enable the development of reproducible therapeutic protocols [1].

In a tendon inflammation model, Pires et al. demonstrated that LLLT at a wavelength of 780 nm (22 mW; 7.7 J/cm²) significantly modulated the expression of inflammatory mediators. In the acute and chronic phases of the process, LLLT decreased the mRNA levels of IL-6, cyclooxygenase 2 (COX-2), and TGF-β while reducing expression of gene encoding TNF-α in the chronic phase [24]. In contrast, Casalechi et al., using a similar model (with LLLT 780 nm, 22 mW; 7.5 J/cm²), demonstrated an increase in the mRNA level of IL-10 and vascular endothelial growth factor (VEGF) and a phase-dependent regulation of levels of MMP-1 and MMP-13, confirming that LLLT can simultaneously modulate the course of the inflammatory process and ECM remodelling [25] .

Despite the effectiveness of LLLT being confirmed in numerous studies, attention is drawn to the significant variability of protocols (wavelength 632**-**1,100 nm; different energy doses 0.6–9.6 J/cm²; exposure durations 16–600 s; number of irradiation sessions) and the lack of a clear standards of irradiation procedure [16, 17, 26, 27]. The heterogeneity of LLLT parameters used by different research groups makes it difficult to clearly determine the relationship between exposure settings and the resulting biological effect, justifying the need for research in strictly controlled conditions. In this context, analysis of the response of fibroblasts cells crucial for repair processes allows for the assessment of the dose-response relationship and the identification of the parameters promoting a pro-regenerative response. The results of our study suggest that the response of fibroblasts to laser irradiation was dependent on the applied parameters. The most favourable effects, reflected by an increased number of adherent metabolically active cells, were observed for the 100 mW/10 J/cm² and 200 mW/2 J/cm² configurations. In the wound healing assay, a parameter-dependent pattern of gap closure dynamics was noted. The most pronounced and sustained effect was observed for cells treated with the 200 mW/2 J/cm² LLLT (Table 2). The fact that only two of the four parameter sets were effective supports the concept of a therapeutic bioenergetic window [27, 28]. This phenomenon is most likely related to the activation of mitochondrial complex IV (cytochrome c oxidase, CCO), leading to an increase in ATP production, activation of the PI3K/Akt pathway and inhibition of NF-κB activity, which promotes the proliferation and migration of fibroblasts, and improves their survival [29, 30].

**Table 2:**
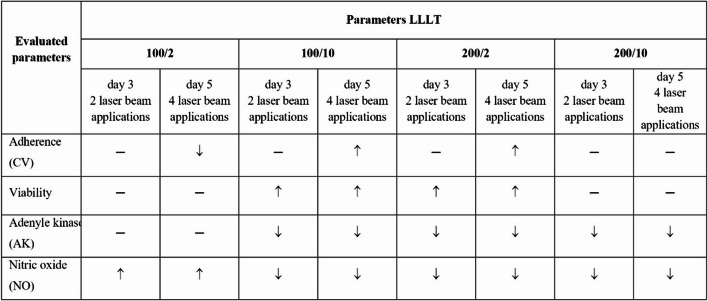
The effect of LLLT on the cellular response of fibroblasts of the Hs680Tr. cell line

The dependence of the observed effect on radiation parameters was confirmed by Anders et al., who demonstrated that irradiation with a wavelength of 810nm (10 mW; 5 J/cm²) increased the mitochondrial metabolism of fibroblasts, while 980 nm (0.2–5 J/cm²) decreased its activity. In turn, in neurons, irradiation with a wavelength of 980 nm induced a dose-dependent effect; low doses (2–200 mJ/cm²) stimulated neurite growth, while higher doses had no pro-regenerative effects [31]. A different response profile was observed in the case of RAW 264.7 macrophages irradiated at a wavelength of 808 nm (100 and 200 mW; 5 and 10 J/cm²), where a pulsed beam of 200 mW and a dose of 5 J/cm² increased cell adhesion, their viability and secretion of NO and TOS, while reduced the levels of TNF-α, monocyte chemoattractant protein-1 (MCP-1/CCL2) and MMP-9 [20]. Brondon et al. also showed that at a wavelength of 670nm (5 J/cm² per treatment; total dose 50 J/cm²), dose fractionation affected the proliferation and metabolism of HEp-2 epithelial cells and L-929 fibroblasts, with the strongest response observed in the case of two exposures per day [32]. In another study of LLLT treatment, a wavelength of 670 nm (5 J/cm²/24 h) and a pulse frequency of 6–600 Hz differentiated the response of HEp-2 cells, with the maximum proliferation being observed at a frequency of 100 Hz [33]. This means that not only the total dose of LLLT, but also the method of its application determines the biological effect.

In numerous studies, wavelength- and dose-dependent effects of LLLT on fibroblast adhesion, migration, and viability have been confirmed. Karimi et al. (660, 808, 915 nm; 150–250 mW; 2 and 4 J/cm²) observed increased adhesion of HGF fibroblasts at 660 and 915 nm, whereas at 808 nm the effect was observed only at 2 J/cm² [34]. Basso et al. demonstrated enhanced migration and proliferation of HGF fibroblasts following irradiation with 780nm laser light (40 mW; 0.5 and 3 J/cm²) [35]. Similarly, Shaikh-Kader et al. reported increased migration of WS1 fibroblasts after exposure to a 660 nm laser beam (100 mW; 5 J/cm²) [36]. Ladiz showed that irradiation at 810 nm (100 mW; 0.5 J/cm²) increased the viability of HGF fibroblasts, whereas 940 nm at the same dose decreased it [37]. In contrast, Harorli et al., irradiating fibroblasts with light at a wavelength of 940nm (40–90 mW; 0.84–1.97 J/cm²), found no effect on cell viability [38]. Collectively, these findings further support the non-linear and parameter-specific character of the LLLT response.

In our study, LLLT not only enhanced ATP-dependent metabolic activity of fibroblasts, but also influenced their membrane integrity and inflammatory status, as evidenced by a decrease in AK and NO release (Table 2). Excessive NO production promotes nitro-oxidative stress and ECM degradation [39, 40]. Therefore, the simultaneous decrease in AK and NO levels may reflect a protective and anti-inflammatory effect of LLLT, potentially associated with inhibition of inducible nitric oxide synthase (iNOS) activity and reduced NF-κB signalling [18]. Together, these findings suggest that mitochondrial activation and chanes in the NO production may represent interconnected mechanisms underlying the observed immunomodulatory effects.

The mechanism of light-mitochondria interaction was described by Karu et al., who demonstrated that NO donors modulated the response of HeLa cells to radiation at a wavelength of 600–860 nm by interacting with cytochrome c oxidase (CCO). At a wavelength of 820 nm, the presence of NO donors abolished the radiation-induced stimulation of the cell adhesion [41, 42]. In the context of our results, this suggests that changes in NO secretion affect mitochondrial activity and that NO signalling may participate in the regulation of LLLT-induced cellular responses.

A decrease in NO secretion was observed by Kocherova et al. during the irradiation of HGF fibroblasts with light at a wavelength of 635nm, whereas no changes were observed at 808 nm using the same power and dose (100 mW, 4 J/cm²) [43]. However, different results were achieved by George et al., who demonstrated an increase in NO secretion following irradiation of primary fibroblasts with a laser beam at 636 and 825 nm wavelengths (power 74 and 104 mW, doses 5–25 J/cm²) [44]. Taken together, these findings suggest that NO modulation is highly sensitive to irradiation parameters and may vary depending on wavelength and dose configuration.

When assessing the potential immunomodulatory effect of LLLT on parameters promoting fibroblast functions associated with tissue regeneration, a decrease in IL-6 and IL-8 secretion was exhibited (Table 3). IL-6 and IL-8 are among factors playing an important role in the healing process [45, 46]. The observed reduction in IL-6 and IL-8 secretion indicates inhibition of fibroblast proinflammatory activity, which may promote the transition from the inflammatory to the proliferative phase of healing. This pattern is consistent with the biphasic dose response characteristic of photobiomodulation.

**Table 3:**
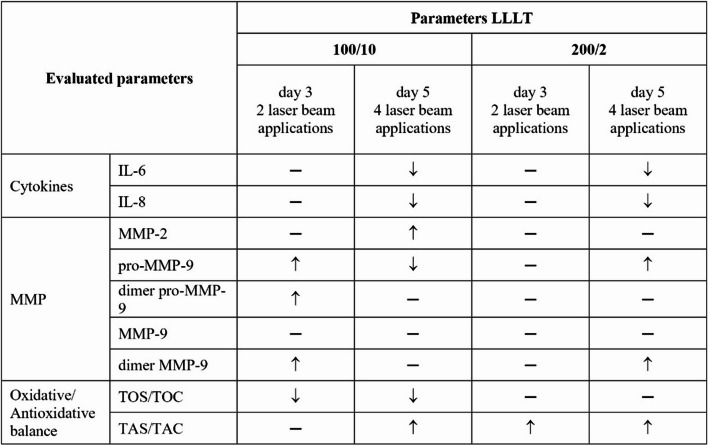
Effect of LLLT on cytokine, MMP activity and oxidative-antioxidative balance in fibroblasts of the Hs680Tr. cell line

Similar results to those reported in the present study were reported by Shaikh-Kader et al., who demonstrated a decrease in IL-6 secretion by WS1 fibroblasts after exposure to light at a wavelength of 660 nm, a power of 100 mW and dose of 5 J/cm² [36]. Harorli et al., in contrast, examined the effect of irradiation of fibroblasts with a beam wavelength of 940nm (power of 40–90 mW, doses of 0.84–1.97 J/cm²), and showed that a dose of 1.97 J/cm² inhibited the release of IL-6 and IL-8 cytokines, while lower doses (0.84–1.4 J/cm²) caused an increase in their secretion [38]. Some authors additionally propose that photobiomodulation may influence inflammatory signalling through epigenetic mechanisms, including microRNA modulation; however, this aspect requires further dedicated mechanistic investigation [38].

Irradiation of fibroblasts with a lower power (100 mW) and a higher dose (10 J/cm²) caused a transient increase (day 3) in pro-MMP-9 and its dimer form, followed by a delayed increase (day five) in MMP-2 activity (Table 3). This indicates controlled, sequential activation of proteolysis, typical of the normal inflammatory and regenerative phases. In contrast, a higher power (200 mW) and lower dose (5 J/cm²) did not affect MMP-2 activity, and the activation of pro-MMP-9 and the MMP-9 dimer was delayed (day five) (Table 3). Thus, the irradiation parameters of 100 mW and 10 J/cm² appear to be more beneficial for the regeneration process as they promote short-term proteolytic activity and subsequent ECM remodelling, reflecting a balanced healing process. These effects may be associated with the modulation of MAPK/ERK and PI3K/Akt pathways involved in MMP regulation, conducted in a manner dependent on the oxidative and inflammatory status of the cells [18, 29, 47].

The decrease in NO and IL-6 and IL-8 cytokine secretion following irradiation observed in our study may indicate that the LLLT parameters used limited excessive inflammatory cell activity, favouring maintenance of a more regenerative rather than proinflammatory environment. In such conditions, MMP-9 activity may occur in a controlled manner, sufficient for early “clearing” of damaged ECM components, but without excessive degradation, while the subsequent increase in MMP-2 activity may reflect the cell transition from the inflammatory phase to that of ECM remodelling. The effect of LLLT on MMP activity has been studied by various authors. Sassoli et al. demonstrated that irradiation of NIH/3T3 fibroblasts with light at a wavelength of 635 nm (power 89 mW, dose 0.3 J/cm²) increased the expression of MMP-2 and MMP-9 . Similarly, Illescas-Montes et al. observed a significant increase in MMP-2 expression for CCD-1064Sk fibroblasts following exposure to 940 nm light (power 0.5 W, dose 4 J/cm²) [48]. Nonetheless, different results were obtained by Yang et al., who observed a decrease in MMP-2 and MMP-9 expression in fibroblasts after the application of 633 nm light (3 mW/cm²) [49].

A significant element of the fibroblast response to LLLT is the reduction of oxidative stress and the increase in antioxidant activity, which promotes redox balance and repair processes. When irradiating fibroblasts with a lower power of 100 mW and dose of 10 J/cm², a decrease was observed in oxidative potential with a simultaneous increase in antioxidant potential. However, applying a power of 200 mW and dose of 2 J/cm², a significant increase was demonstrated in antioxidant potential (Table 3). These results suggest that appropriately selected LLLT parameters can promote the maintenance of oxidative-antioxidant balance in a manner conducive to tissue regeneration. The mechanism of this phenomenon may be related to the activation of the Nrf2 pathway and the increased expression of antioxidant enzymes such as SOD and GPx, while limiting the overproduction of reactive oxygen species (ROS) [50, 51]. 

Similar observations were presented by George et al., who, using light at a wavelength of 636nm and 825 nm (power of 74 and 104 mW, doses of 5–25 J/cm², respectively), showed that the 636 nm wave reduced the secretion of ROS, while the 825 nm wave intensified their production [44]. Baldassarro et al. demonstrated that light at a wavelength of 645 nm (power of 11 mW) reduces the oxidative potential while increasing the antioxidant potential of fibroblasts [52].

Cells were cultured for a specified number of days and irradiated with a pulsed laser beam (frequency 100 Hz, 50% duty cycle) at a wavelength of 808 nm, with a power output of 100 or 200 mW and energy doses of 2–10 J/cm² per well containing cells (groups: 100/2, 100/10, 200/2, 200/10). On days 3 and 5 of the experiment, the following parameters were measured: number of adherent cells (CV), number of metabolically active cells, adenylate kinase (AK) level, and nitric oxide (NO) level. Statistically significant differences between the irradiated groups (with various laser parameters) and the non-irradiated control group were indicated by arrows, where ↑ denotes an increase, ↓ denotes a decrease, and ‒ denotes no statistically significant differences.

Cells were cultured for a specified number of days and irradiated with a pulsed laser beam (frequency 100 Hz, 50% duty cycle) at a wavelength of 808 nm, with a power output of 100 mW and an energy dose of 10 J/cm² per well containing cells, as well as with a power output of 200 mW and an energy dose of 2 J/cm² per well containing cells (groups: 100/10 and 200/2). On days 3 and 5 of the experiment, the levels of cytokines (IL-6, IL-8, IL-1β, IL-10, IL-12p70, TNF-α), metalloproteinases (MMP-2, pro-MMP-9, MMP-9), and the oxidative (TOS/TOC) and antioxidative (TAS/TAC) potential of the cells were determined. Statistically significant differences between the irradiated groups (with different laser parameters) and the non-irradiated control group were indicated by arrows, where ↑ denotes an increase, ↓ denotes a decrease, and - denotes no statistically significant differences.

## Conclusion

We confirmed that the fibroblast response to LLLT is closely dependent on the irradiation parameters. In the analysed cellular model, the most beneficial effects were obtained when cells were irradiated with parameters at 200 mW; 2 J/cm² and 100 mW; 10 J/cm². The results indicate that appropriately selected radiation settings can promote regulation of inflammatory activity, extracellular matrix remodelling and oxidant-antioxidant balance, enabling the development of a pro-regenerative functional profile of cells *in vitro.*

## Limitations

The experiments were performed independently in triplicate; however, they were conducted within the same fibroblast cell line, meaning that the biological model was limited to a single cell source. Consequently, this may affect the generalizability of the obtained results and limit their interpretation in a broader biological context. Furthermore, the in vitro monoculture model does not reflect the complex intercellular interactions involved in the healing process. Therefore, future studies should include both independent biological models and more complex experimental systems, including co-culture models and in vivo studies, to determine whether the selected LLLT parameters produce effects at the tissue and whole-organism levels. 

## Supplementary Information

Below is the link to the electronic supplementary material.

Supplementary Material 1 (PDF 213 KB)

Supplementary Material 2 (PDF 327 KB)

## Data Availability

The data that support the findings of this study are available from the corresponding author upon reasonable request.

## Declarations

### Clinical trial number

Not applicable. The study was conducted in vitro using a commercially available established cell line purchased from a certified cell bank.

## Abbreviations

LLLT: Low–level laser therapy
AK: Adenylate kinase
NO: Nitric oxide
MMP: Metalloproteinase
IL: 6–interleukin 6
IL: 8–interleukin 8
ECM: Extracellular matrix
TNF: α–tumor necrosis factorα
TGF: β–transforming growth factorβ
IL: 10–interleukin 10
PBM: Photobiomodulation
CCO: Cytochrome c oxidase
ROS: Reactive oxygen species
Akt: Protein kinase B
ERK: Extracellular signal–regulated kinase
JNK: c–Jun N–terminal kinase
CV: Crystal violet
PF: Paraformaldehyde
IL: 8–interleukin 8
IL: 12p70–interleukin 12p70
SDS: Sodium dodecyl sulphate
TOS/TOC: Total oxidative status
TOS: Total oxidative capacity
TAS/TAC: Total antioxidant status
COX: 2–cyclooxygenase 2
VEGF: Vascular endothelial growth factor
MCP: 1/CCL2–monocyte chemoattractant protein–1
iNOS: Inducible nitric oxide synthase
