# Supplementary material for: Optimisation of low-level light therapy (lllt) parameters and evaluation of its immunomodulatory effect on fibroblasts
Source: Lasers Med Sci. 2026 May 9;41(1):89. doi: 10.1007/s10103-026-04873-w (PMC13156211; doi:10.1007/s10103-026-04873-w)

3 day

1 2 3 4

CTR sample numbers 13-16

Groups

100/10 sample numbers 25-28

200/2 sample numbers 36-39

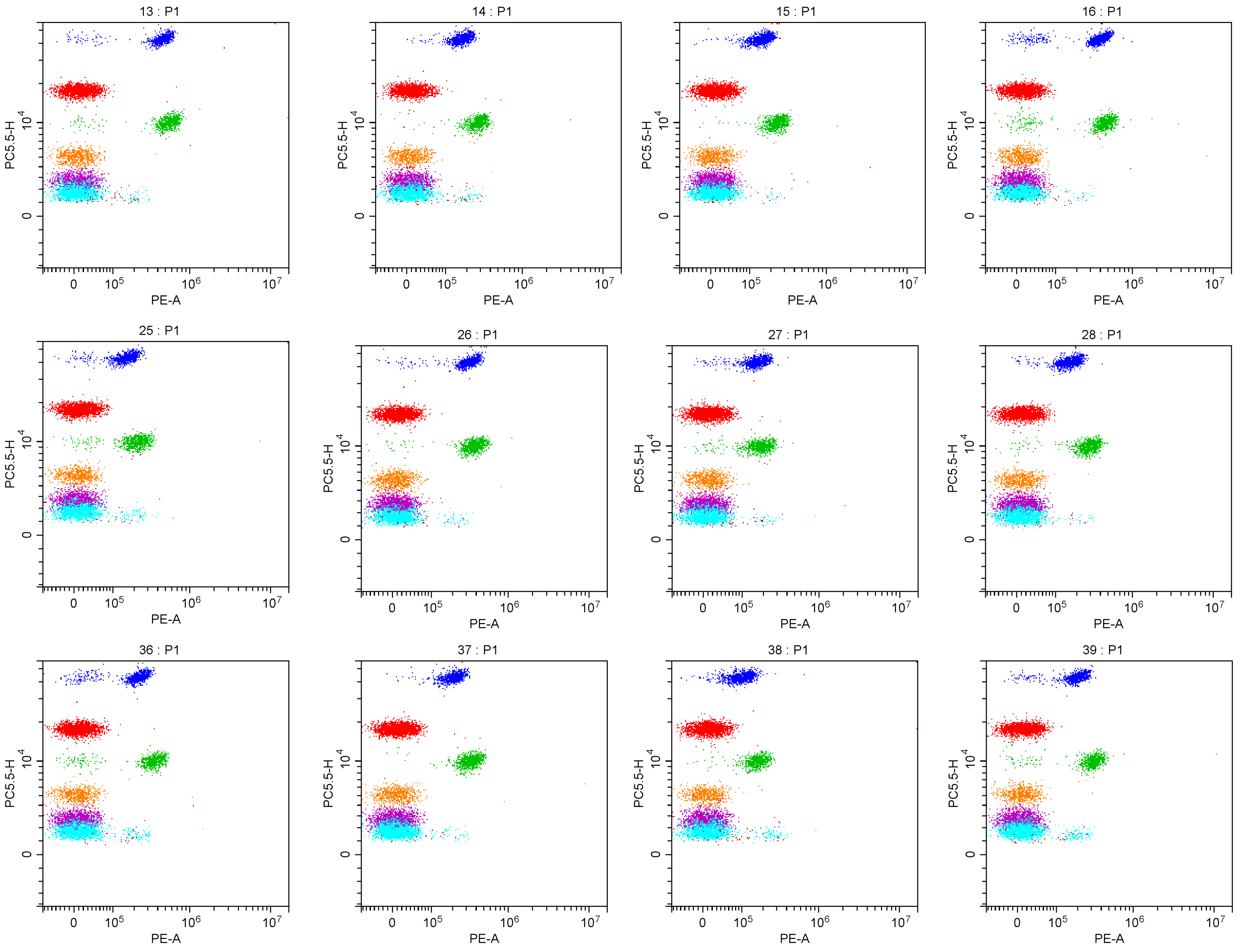

5 day

1 2 3 4

Groups

CTR sample numbers 48-51

100/10 sample numbers 60-63

200/2 sample numbers 72-75

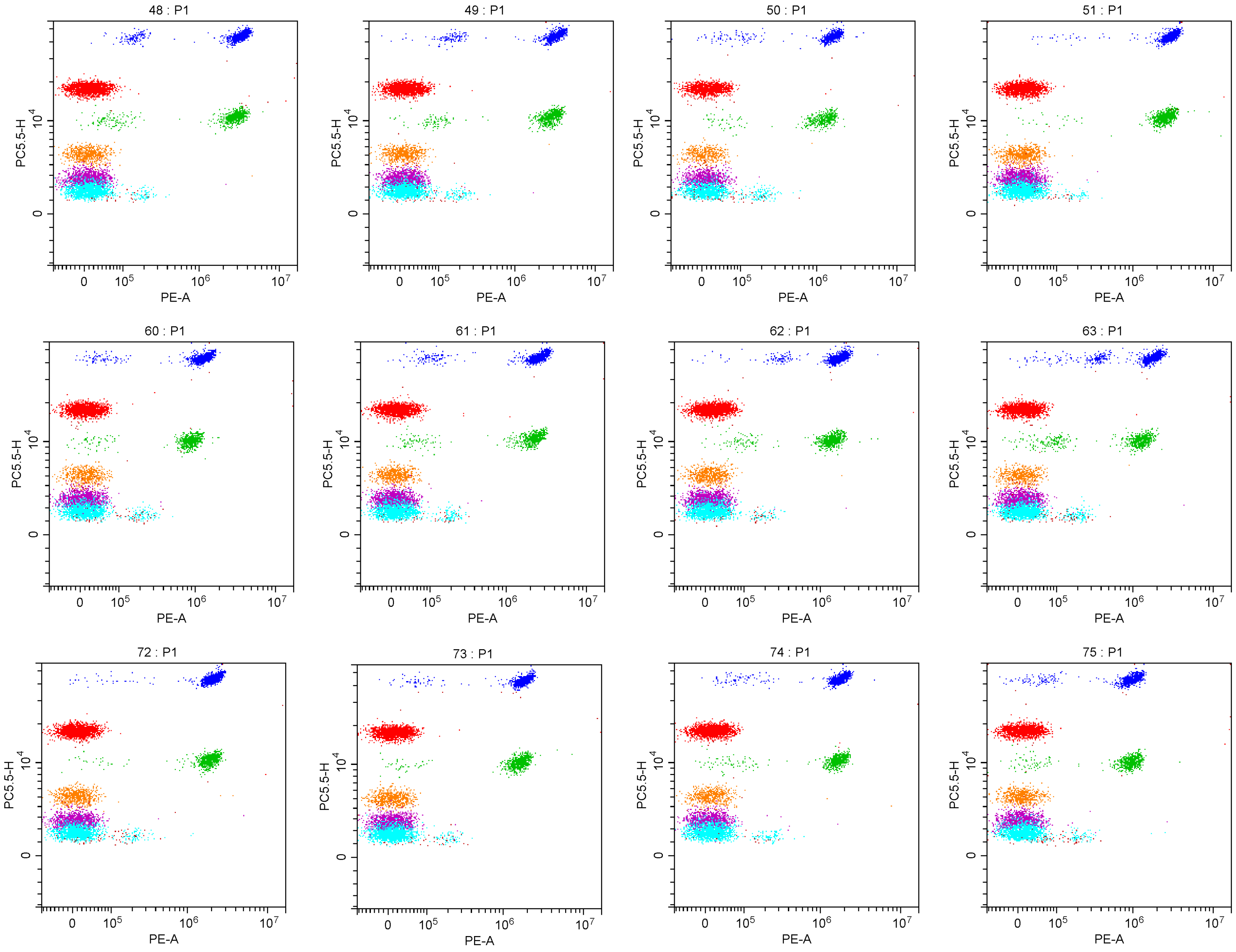

Supplement: Supplementary file 1 — Supplementary Material 1 (PDF 213 KB) [file 10103_2026_4873_MOESM1_ESM.pdf]
