# Supplementary material for: Optimisation of low-level light therapy (lllt) parameters and evaluation of its immunomodulatory effect on fibroblasts
Source: Lasers Med Sci. 2026 May 9;41(1):89. doi: 10.1007/s10103-026-04873-w (PMC13156211; doi:10.1007/s10103-026-04873-w)
Supplement: Supplementary file 2 — Supplementary Material 2 (PDF 327 KB) [file 10103_2026_4873_MOESM2_ESM.pdf]

Zymography gels presented in this study include lanes originating both from the experiments described in the manuscript and from parallel studies conducted under similar conditions within the same research project.For clarity, only the lanes marked with specific identifiers correspond to the data analyzed and interpreted in this work. The additional lanes, although visible in the gel images, were not used in the preparation of this manuscript and do not contribute to the reported findings.

In certain gelatin zymography gels, visible bands were observed; however, densitometric analysis could not be performed for all samples due to limited detection sensitivity. This limitation is likely attributable to the low concentration of active metalloproteinases under the in vitro conditions applied, which fell below the threshold required for reliable quantification using this method. To improve detection in future experiments, it may be necessary to increase the sample volume or employ a more sensitive technique.

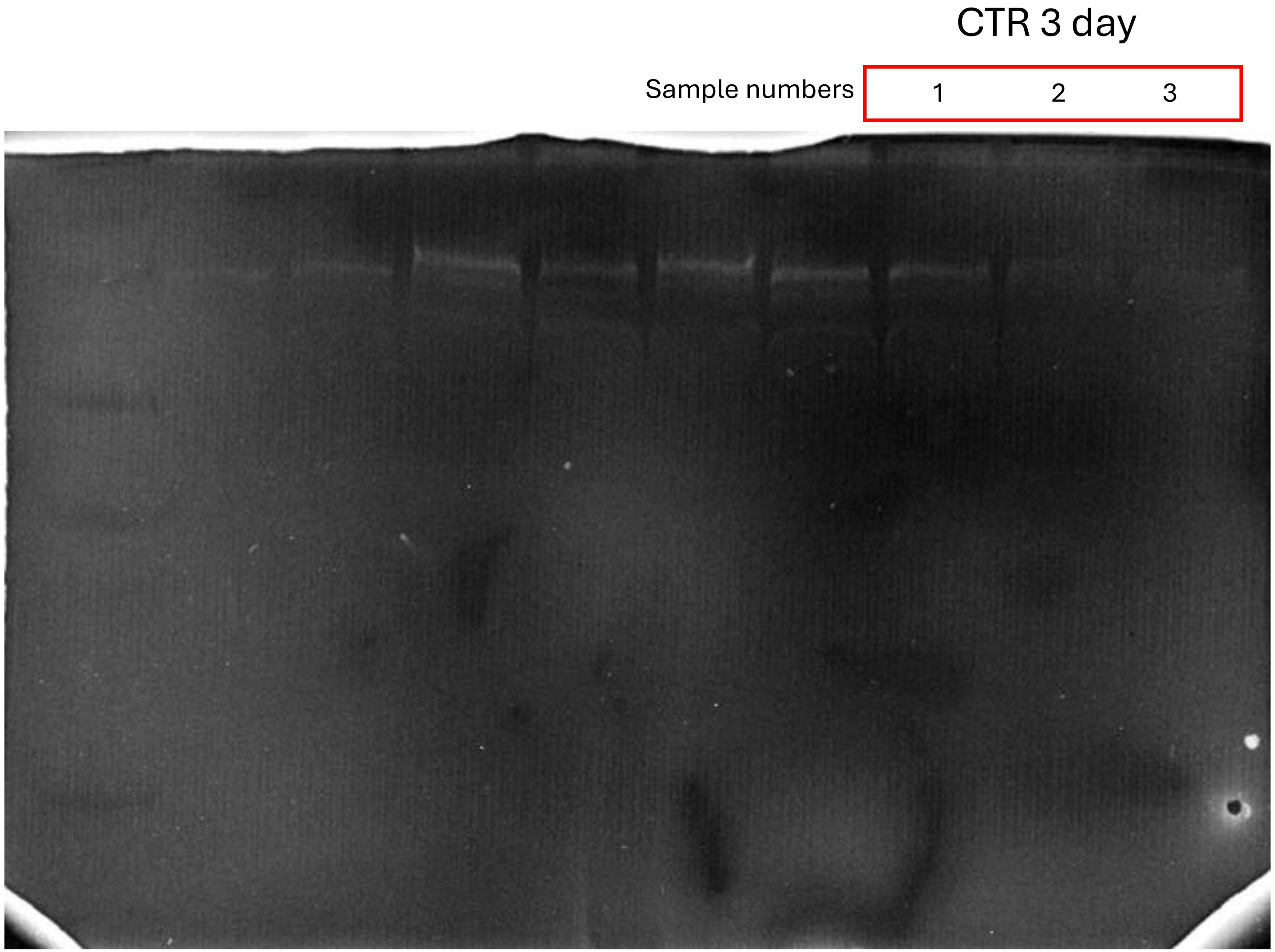

The lanes labeled with specific numbers correspond to the data presented in this manuscript. The remaining lanes originate from parallel experiments not included in the study and are shown for technical completeness.

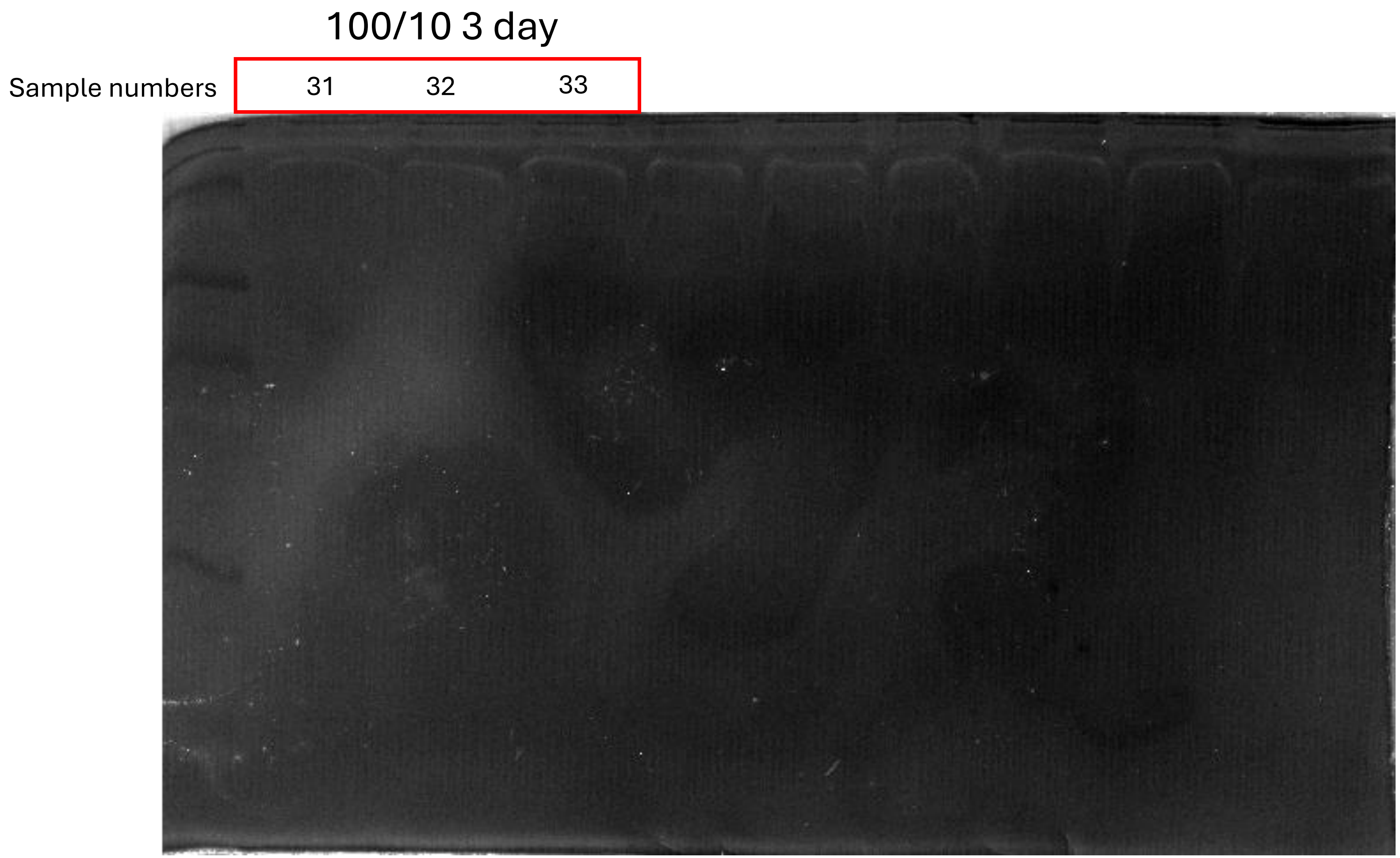

The lanes labeled with specific numbers correspond to the data presented in this manuscript. The remaining lanes originate from parallel experiments not included in the study and are shown for technical completeness.

200/2 3 day

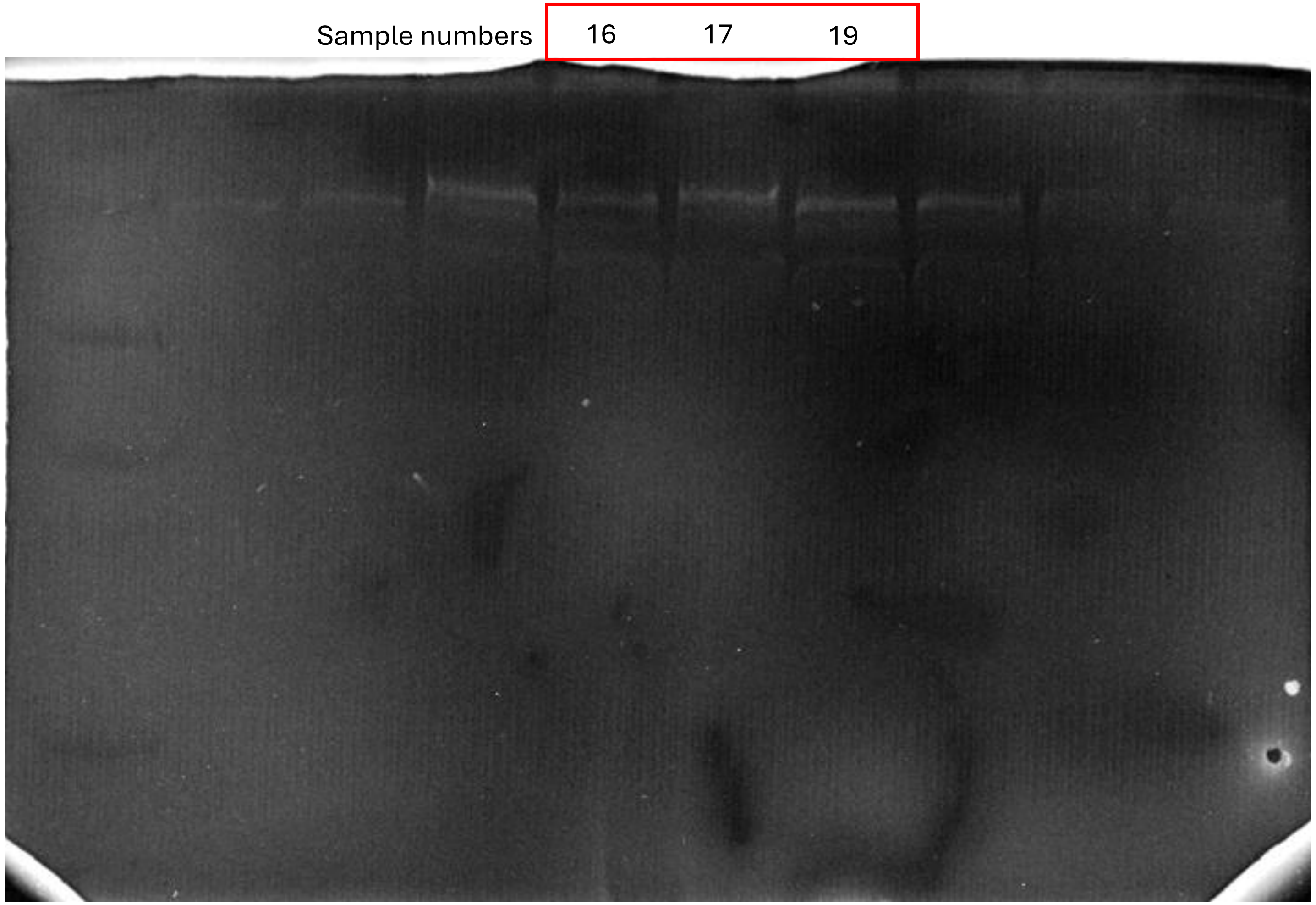

The lanes labeled with specific numbers correspond to the data presented in this manuscript. The remaining lanes originate from parallel experiments not included in the study and are shown for technical completeness.

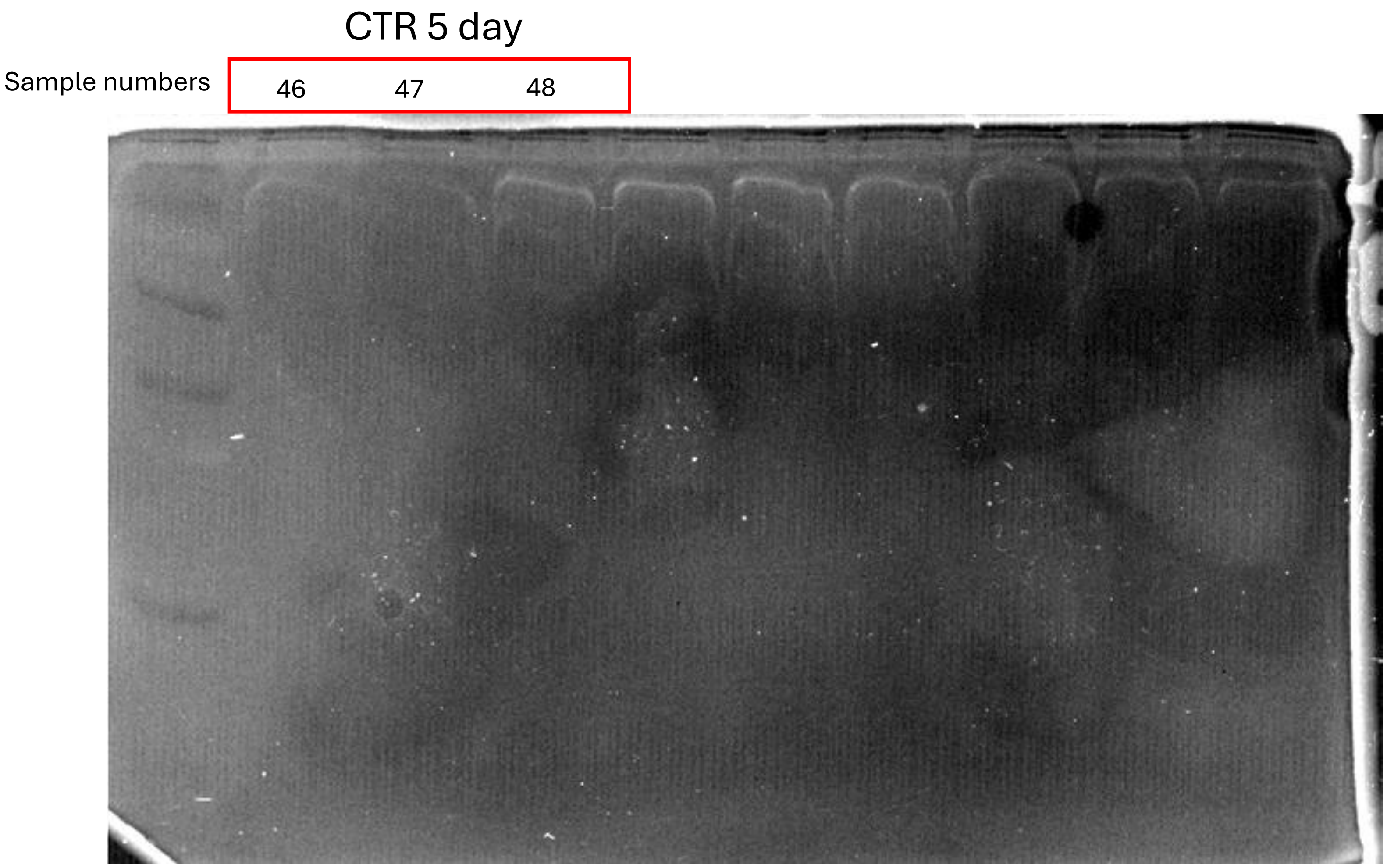

The lanes labeled with specific numbers correspond to the data presented in this manuscript. The remaining lanes originate from parallel experiments not included in the study and are shown for technical completeness.

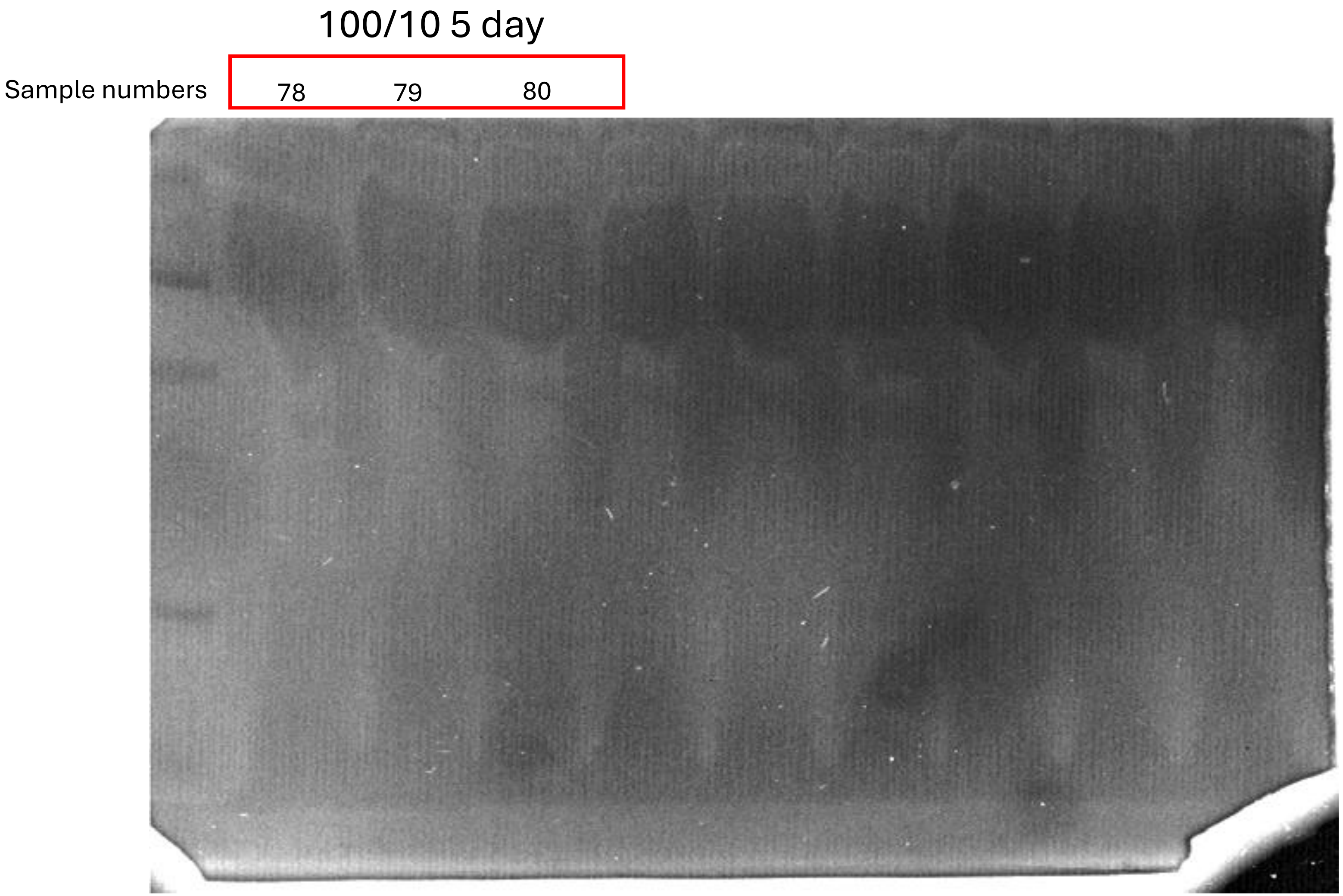

The lanes labeled with specific numbers correspond to the data presented in this manuscript. The remaining lanes originate from parallel experiments not included in the study and are shown for technical completeness.

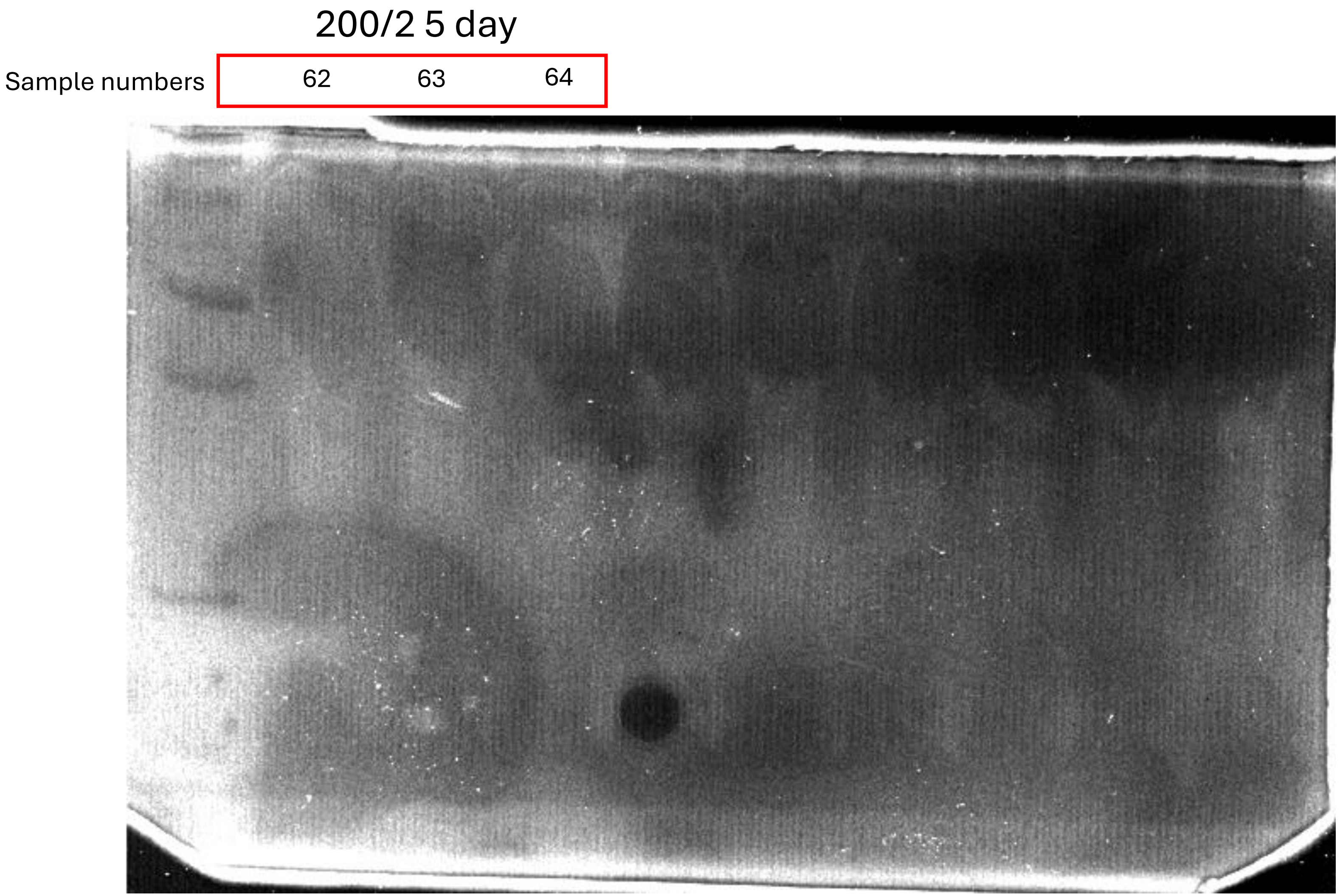

The lanes labeled with specific numbers correspond to the data presented in this manuscript. The remaining lanes originate from parallel experiments not included in the study and are shown for technical completeness.
